# Association between late afternoon and early evening physical activity and next-day depression and anxiety symptoms among workers in Japan: a two-week longitudinal study

**DOI:** 10.1186/s12889-026-28187-2

**Published:** 2026-06-18

**Authors:** Yuki Shimada, Kazuhiro Watanabe, Hiroyuki Hikichi, Keiichi Matsuzaki, Akizumi Tsutsumi

**Affiliations:** 1 Undergraduate, Kitasato University School of Medicine, 1-15-1 Kitazato, Minami-ku, Sagamihara, 252-0374 Japan; 2 Department of Public Health, Kitasato University School of Medicine, 1-15- 1 Kitazato, Minami-ku, Sagamihara, 252-0374 Japan; 3 Department of Digital Mental Health, Graduate School of Medicine, The University of Tokyo, 7-3-1 Hongo, Bunkyo-ku, Tokyo, 113-8655 Japan

**Keywords:** Mental health, Workplace, Behavioral medicine, Prevention, Longitudinal study, Digital health, Mobile health

## Abstract

**Background:**

While physical activity is a preventive factor against depression and anxiety, less is known about how the timing of physical activity throughout the day is associated with improved mental health the next day among workers. This study investigated the association between physical activity in the late afternoon and early evening and next-day symptoms of depression and anxiety among workers.

**Methods:**

We conducted a secondary analysis of a two-week longitudinal study. Participants were full-time, daytime workers employed in either the private or public sector, residing or working in urban areas of Japan, and owning a personal smartphone. Participants were instructed to install Google Fit app to passively track physical activity. They also completed daily web-based surveys between 5:00 PM and 6:00 PM, using the Japanese K6 scale to assess depression and anxiety symptoms. Physical activity was categorized into two timeframes: late afternoon (3:00 PM to 6:00 PM) and early evening (6:00 PM to 9:00 PM). A K6 score ≥ 5 was classified as a high symptom level for depression and anxiety. Associations were analyzed using generalized linear mixed models, adjusting for age, gender, and previous-day depression and anxiety symptoms.

**Results:**

A total of 116 participants met the eligibility criteria, contributing 1,400 observation days. A high symptom level for depression and anxiety was reported on 259 days (18.5%). Physical activity in the late afternoon showed a non-significant negative association with next-day depression and anxiety symptoms (OR = 1.00, 95% CI: 0.99–1.01, *p* = 0.472). In contrast, early evening activity indicated a marginally significant association with lower symptoms (OR = 0.99, 95% CI: 0.97–1.00, *p* = 0.061). Regarding the covariates, a higher K6 level on the previous day and younger age (≤ 39 years) were significant predictors. Subgroup analyses showed significant negative associations for having low levels of depression and anxiety symptoms on the previous day (K6 score < 5) (OR = 0.98, 95% CI: 0.96–0.997, *p* = 0.023) during this timeframe.

**Conclusions:**

Physical activity in the early evening was associated with reduced risk of next-day depression and anxiety symptoms, particularly when prior-day symptom levels were lower.

**Supplementary Information:**

The online version contains supplementary material available at 10.1186/s12889-026-28187-2.

## Background

According to data from the Global Burden of Disease, approximately 15% of workers experience mental health conditions, such as depression and anxiety, during employment [1]. These mental health issues among workers are crucial risk factors for non-communicable diseases [2–4]. In addition, a global return-on-investment analysis across 36 countries estimated that workers’ mental health problems led to over 12 billion absences from work, resulting in a 925 billion USD loss [5]. Therefore, preventing mental health problems at work is a significant issue to maintain employees’ well-being as well as their work efficiency.

Physical activity is a well-established preventive factor against depression and anxiety among non-clinical and generally healthy populations [6]. Individuals who engage in higher levels of physical activity have a lower risk of developing depression compared to those with lower activity levels [7]. Not only habitual aerobic activities but also various forms and intensities of physical activity, including resistance training [8] and walking less than 150 min per week [9], have been shown to lower the risk of subsequent depression. A previous review suggested that its antidepressant effects are mediated through a combination of biological mechanisms, including the stimulation of neuroplasticity via increased brain-derived neurotrophic factor levels, the reduction of systemic inflammation and oxidative stress, and the normalization of hypothalamic-pituitary-adrenal axis activity [10]. Additionally, physical activity positively impacts sleep quality and duration [11, 12], which may serve as mediators in its effect on mental health. Based on these benefits, promoting physical activity is widely recommended to prevent mental health conditions at the workplace [13, 14].

Several studies have explored short-term or daily associations between physical activity and mental health outcomes. A systematic review utilizing the experience sampling method concluded that physical activity predicts subsequent reductions in depressed mood [15]. Experimental studies have also demonstrated immediate improvements in mood following morning or evening exercises among healthy participants [16, 17]. Evening physical activity, in particular, has been associated with longer sleep duration and better sleep quality on the same night [18, 19]. A randomized crossover study highlighted that exercising approximately four hours before habitual bedtime significantly improves sleep quality [20]. Given that sleep is a key determinant of mental health, physical activity may have cross-day effects on mental health outcomes the next day. However, less is known about how the timing of physical activity throughout the day is associated with improved mental health the next day among workers. Most previous research has focused on patients, university students, or community residents, limiting its applicability to the working population. Clarifying these cross-day associations in working populations could inform more targeted recommendations regarding the optimal timing of physical activity to support mental health.

Therefore, the present study aimed to investigate the association between physical activity during specific timeframes and symptoms of depression and anxiety on the following day among workers. Specifically, we focused on the amount of physical activity during the late afternoon (3:00 PM to 6:00 PM) and early evening (6:00 PM to 9:00 PM). We utilized a smartphone accelerometer app, Google Fit, to monitor participants’ physical activity in a real-world, free-living setting. Smartphones used as stand-alone monitors provide estimates that are comparable to those obtained from research-grade accelerometers and may serve as a feasible alternative [21, 22], although they tend to underestimate activity during short or intermittent bouts [23]. We hypothesized that greater physical activity during these timeframes would be associated with lower risks of depression and anxiety symptoms the next day.

## Methods

### Study design and setting

This study was a secondary analysis of data from a two-week longitudinal study originally conducted to develop a deep learning model for the passive monitoring of depression and anxiety symptoms among workers [24]. The online survey was conducted sequentially over two weeks between November 2021 and April 2022 to collect digital data on physical activity and assess daily psychological distress. Participants were recruited from private companies in the Kanto region of Japan and through the social networking service X (formerly Twitter). The survey was developed for this longitudinal study (Appendix 1). After providing informed consent and completing a baseline survey assessing demographic information and initial psychological distress, participants were instructed to download and install Google Fit app on their smartphones to track their physical activity. During the observation period, participants received a daily questionnaire, delivered between 5:00 PM and 6:00 PM, to assess psychological distress. At the end of the study, participants were asked to export and submit their Google Fit data to the research team. The study protocol was approved by the Kitasato University Medical Ethics Organization (B21-119). This manuscript adheres to the guidelines of the Strengthening the Reporting of Observational Studies in Epidemiology (STROBE) statement [25].

### Participants

The original longitudinal study [24] included the workers who (1) were employed in a public- or private-sector organization, (2) resided or worked in urban areas of Japan, and (3) owned a personal smartphone. Workers who were absent at baseline were excluded. For the present analysis, additional inclusion criteria were applied: (4) being a full-time/regular employee and (5) working day shifts.

### Measurements

#### Depression and anxiety symptoms

Depression and anxiety symptoms were assessed daily using the Japanese version of the Kessler Psychological Distress Scale (K6) [26]. The K6 is a self-administered questionnaire that screens for mental health conditions. It consists of six items assessing the frequency of symptoms related to depression and anxiety, each rated on a five-point Likert scale ranging from 0 (none of the time) to 4 (all the time). Higher total scores indicated a greater risk of depression and anxiety symptoms. The reliability and validity of the Japanese version have been confirmed [26]. In this study, based on established studies, a K6 score ≥ 5 indicated a high symptom level for depression and anxiety [27].

#### Physical activity

Physical activity data were digitally collected using Google Fit app, which recorded activity every 15 min over a 24-hour period, and categorized it by intensity (light, moderate, or vigorous). Detailed information on data collection and reliability is provided by Google [28]. For this study, we used the total duration of physical activity across all intensity levels during specific timeframes as a continuous variable.

#### Demographic variables

We collected demographic information including age (< 20, 20–29, 30–39, 40–49, 50–59, 60–69, and ≥ 70 years) and gender (men, women, other, and no response). Additional data were collected on occupation (management; engineering or education; general office tasks; sales; services; transportation; construction; production or skilled labor; and agriculture, forestry, or fisheries) and average working hours per week during the previous month (1–34, 35–40, 41–50, 51–60, 61–65, 66–70, and ≥ 71 h). We also recorded whether each survey response occurred on a working or non-working day.

### Sample size calculation

As this study was a secondary analysis of existing data, no a priori sample size calculation was performed to determine the required sample size for detecting associations between time-specific physical activity and depression or anxiety. Instead, a post-hoc power analysis was conducted after completing the primary analyses. This power analysis was performed using the “simr” package in R (version 4.4.2).

### Statistical analysis

We extracted data that met the inclusion criteria and summarized the participants’ demographic characteristics. Based on the timeframe definitions provided by the Japan Meteorological Agency [29], physical activity duration was aggregated into two timeframes: 3:00 PM to 6:00 PM (late afternoon) and 6:00 PM to 9:00 PM (early evening). A K6 score ≥ 5 was used to indicate a high symptom level for depression and anxiety on the following day, in accordance with established cut-off values [27]. To examine the simple bivariate association between physical activity and next-day symptoms of depression and anxiety, we compared the proportion of observations with a high symptom level for depression and anxiety (K6 score ≥ 5) across categories of physical activity duration in the two timeframes (late afternoon and early evening). Physical activity duration was categorized into seven groups: <10, 10–19, 20–29, 30–39, 40–49, 50–59, and ≥ 60 min. Chi-squared tests were conducted to assess whether the proportion of a high symptom level for depression and anxiety differed across these physical activity duration groups. Following this, for the primary analysis, we employed a generalized linear mixed model (binomial distribution with a logit link function) to examine associations between physical activity in the two timeframes and next-day depression and anxiety symptoms. The model was adjusted for age, gender, and depression/anxiety levels on the previous day. Age was categorized into three groups: ≤ 39 years, 40–49 years (reference), and ≥ 50 years. Gender was categorized into two groups (men [reference] and women), as there were no respondents in the “other” or “no response” categories. Other demographic variables (occupations, working hours, and whether the day was a working or non-working day) were excluded from the model due to convergence issues. Given that the dataset included repeated measures within the individuals, a random intercept was included in the model. Additionally, sensitivity analyses were conducted to examine variations in risks for depression and anxiety symptoms using physical activity thresholds of 10, 20, 30, 40, 50, and 60 min within each timeframe. Each threshold was tested in a separate analysis. Furthermore, an additional sensitivity analysis was conducted to examine whether the association varied according to baseline symptom levels. The analysis excluded days with extremely high symptom levels (K6 score ≥ 13) [27] on the previous day, as individuals with such severe conditions were less likely to engage in physical activity. Subgroup analyses were also conducted by classifying days according to symptom levels on the previous day, defined as low (K6 score < 5) and high (K6 score ≥ 5). These models were adjusted by age and gender categories to estimate the associations.

Missing values in the K6 scores were imputed using multiple imputation (m = 50) via weighted predictive mean matching. All analyses were performed in R (version 4.4.2), using the “lme4” package for generalized linear mixed models and the “mice” package for multiple imputation.

## Results

### Participant characteristics

**Fig. 1:**
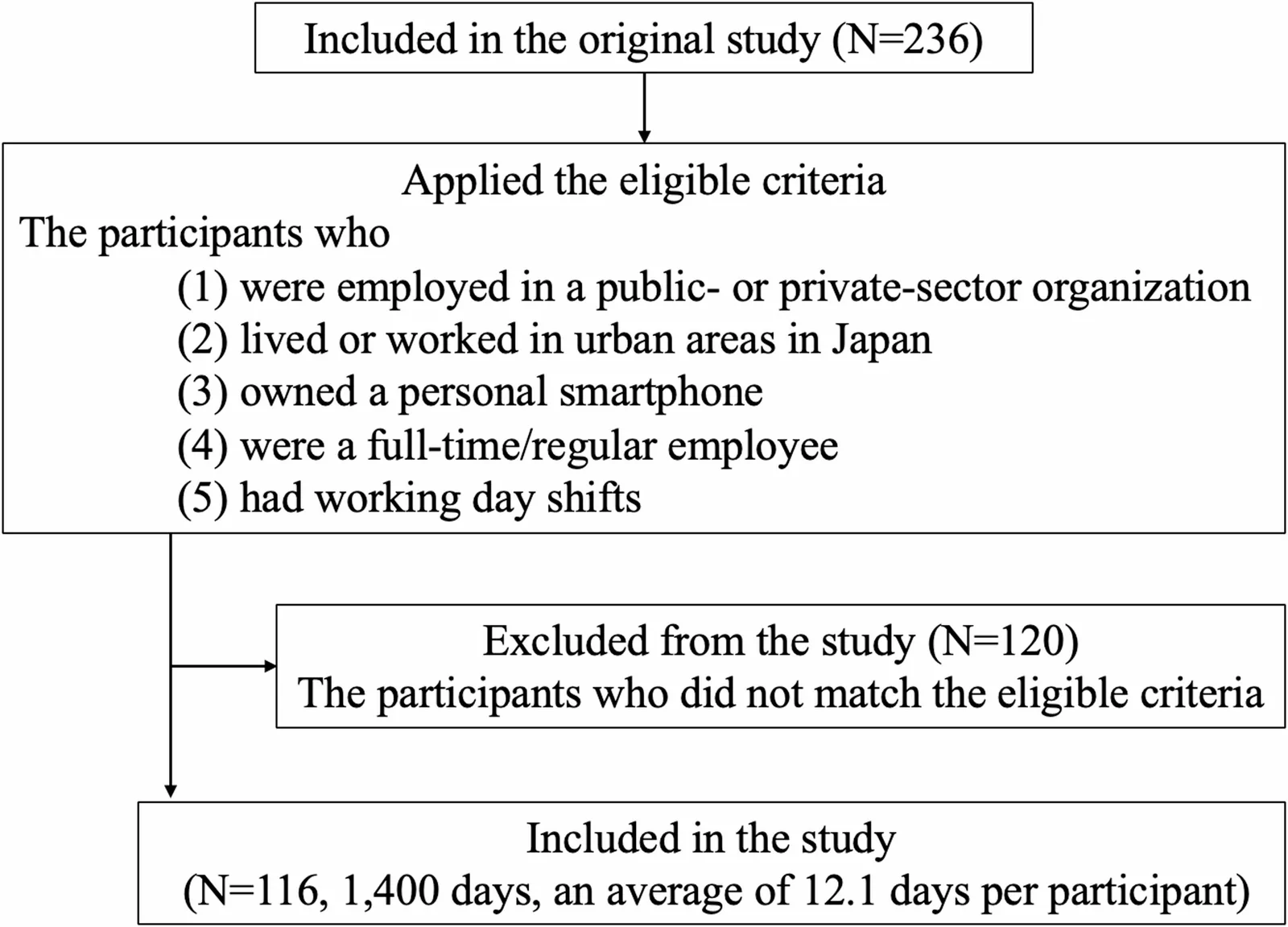
Participant flowchart

Of the 236 workers who participated in the original study, 116 met the eligibility criteria for the present analysis (Fig. 1). The 116 participants provided Google Fit data covering a total of 1,400 observation days, with an average of 12.1 days per participant. Among all observation days, 892 days (63.7%) were workdays and 399 days (28.5%) were non-workdays. Information was missing for the remaining 109 days.

Table 1 summarizes the demographic characteristics of the participants and descriptive statistics of physical activity and depression and anxiety symptoms. The largest age group was 40–49 years (31%). A high symptom level for depression and anxiety, defined as a K6 score of ≥ 5, was reported on 259 days (18.5%) out of the total 1,400 observation days. Missing values for K6 scores were identified on 109 days and imputed accordingly. The mean durations of physical activity during the late afternoon and early evening ranged from 12.18 to 13.61, with large variances among the observation days. The mean K6 score was 2.66, also indicating substantial variability.

**Table 1:** Characteristics of the participants (*n* = 116) and descriptive statistics of PA and depression/anxiety symptoms

Characteristics	Participants, *n* (%)	Mean (SD)	Median (IQR)
Age (years)			
< 20	0 (0)	—	—
20–29	19 (16)	—	—
30–39	34 (29)	—	—
40–49	36 (31)	—	—
50–59	24 (21)	—	—
60–69	3 (3)	—	—
≥ 70	0 (0)	—	—
Gender			
Men	66 (57)	—	—
Women	50 (43)	—	—
Others or no response	0 (0)	—	—
Occupation			
Manager	27 (23)	—	—
Professional/engineer/academic	36 (31)	—	—
Clerk	33 (28)	—	—
Sales	12 (10)	—	—
Services	5 (4)	—	—
Transportation	0 (0)	—	—
Construction	0 (0)	—	—
Production/Skilled	2 (2)	—	—
Agriculture/Forestry/Fisheries	0 (0)	—	—
Others	1 (1)	—	—
Weekly working hours (h)			
1–34	4 (3)	—	—
35–40	30 (26)	—	—
41–50	58 (50)	—	—
51–60	20 (17)	—	—
61–65	2 (2)	—	—
66–70	1 (1)	—	—
≥ 70	1 (1)	—	—
Physical activity			
Late afternoon (3:00 PM to 6:00 PM)			
Moderate + Vigorous (minutes)	—	12.18 (21.2)	2.00 (14.3)
Vigorous (minutes)	—	2.48 (8.0)	0.00 (0.0)
Early evening (6:00 PM to 9:00 PM)			
Moderate + Vigorous (minutes)	—	13.61 (20.4)	5.00 (21.0)
Vigorous (minutes)	—	3.75 (9.6)	0.00 (4.0)
Depression and anxiety symptoms (K6 score)	—	2.66 (4.2)	3.00 (3.0)

*SD* standard deviation, *IQR* interquartile range, *PA* physical activity, *K6* the Kessler Psychological Distress Scale

### Association between physical activity and depression and anxiety symptoms in the specific timeframes

Table 2 presents the bivariate associations between physical activity and next-day depression and anxiety symptoms during the late afternoon and early evening. In both timeframes, physical activity durations were significantly associated with the presence of a high symptom level for depression and anxiety (*p* = 0.017 for late afternoon; *p* = 0.012 for early evening). Standardized residuals from the chi-squared tests revealed that the highest activity group (≥ 60 min) had significantly fewer days with the symptoms in both timeframes. Additionally, in the early evening timeframe, the lowest activity group (< 10 min) showed significantly more days with a high symptom level for depression and anxiety.

**Table 2:** The bivariate association between physical activity and next-day depression/anxiety symptoms in the two timeframes

Late afternoon (3:00 PM to 6:00 PM)									
Physical activity duration	< 10 min	10–19 min	20–29 min	30–39 min	40–49 min	50–59 min	≥ 60 min	Total observation	p for difference
	(849 days)	(170 days)	(91 days)	(56 days)	(33 days)	(21 days)	(71 days)	(1291 days)	
	n (%)	n (%)	n (%)	n (%)	n (%)	n (%)	n (%)	n (%)	
K6 < 5	674 (79.4)	137 (80.6)	71 (78.0)	42 (75.0)	22 (66.7)	20 (95.2)	66 (93.0)	1032 (79.9)	0.017
K6 ≥ 5	175 (20.6)	33 (19.4)	20 (22.0)	14 (25.0)	11 (33.3)	1 (4.8)	5 (7.0)	259 (20.1)	
Early evening (6:00 PM to 9:00 PM)									
Physical activity duration	< 10 min	10–19 min	20–29 min	30–39 min	40–49 min	50–59 min	≥ 60 min	Total observation	p for difference
	(763 days)	(176 days)	(162 days)	(82 days)	(42 days)	(28 days)	(38 days)	(1291 days)	
	n (%)	n (%)	n (%)	n (%)	n (%)	n (%)	n (%)	n (%)	
K6 < 5	590 (77.3)	141 (80.1)	139 (85.8)	68 (82.9)	32 (76.2)	25 (89.3)	37 (97.4)	1032 (79.9)	0.012
K6 ≥ 5	173 (22.7)	35 (19.9)	23 (14.2)	14 (17.1)	10 (23.8)	3 (10.7)	1 (2.6)	259 (20.1)	

Missing values for K6 scores were identified on 109 days

*K6* the Kessler Psychological Distress Scale

**Fig. 2:**
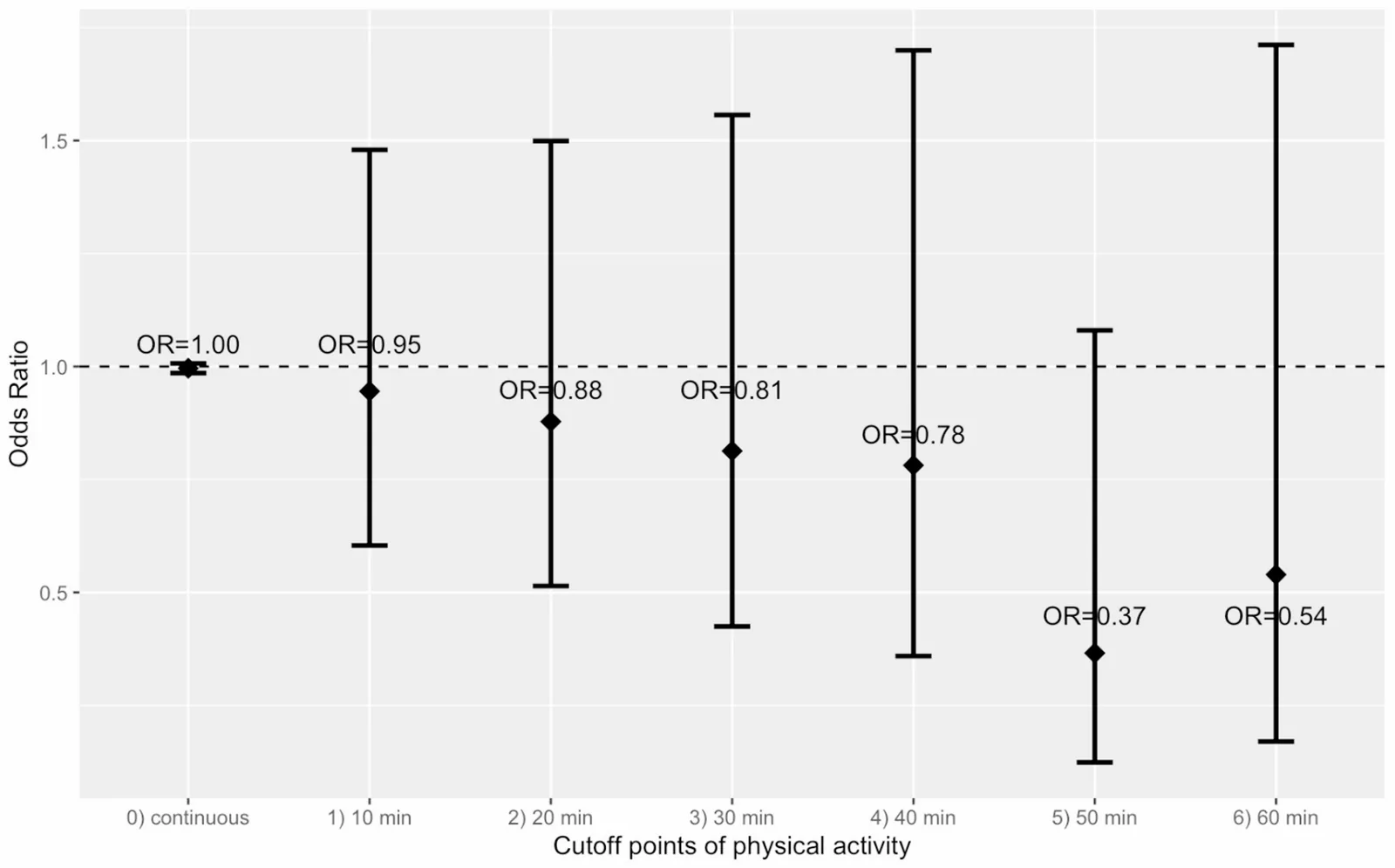
Sensitivity analysis results of physical activity in the late afternoon (3:00 PM to 6:00 PM)

Table 3 presents the results of a generalized linear mixed model, which indicates the association between late afternoon physical activity and next-day depression and anxiety symptoms. In the crude model, a negative but non-significant association was observed (odds ratio [OR] = 0.99; 95% confidence interval [CI]: 0.98–1.01; *p* = 0.294). The adjusted model similarly showed a non-significant negative association (OR = 1.00; 95% CI: 0.99–1.01; *p* = 0.472). Regarding the covariates, a higher K6 level on the previous day (OR = 5.10; 95% CI: 2.99–8.72; *p* < 0.001) and younger age (≤ 39 years) (OR = 3.06; 95% CI: 1.09–8.57; *p* = 0.034) were significantly associated with the outcome. Sensitivity analysis results for this timeframe are illustrated in Fig. 2. Although none of the models reached statistical significance, a trend was observed in which longer durations of physical activity were associated with lower ORs for a high symptom level for depression and anxiety. No other additional analyses yielded statistically significant results.

**Table 3:** Multivariate association between physical activity and next-day depression/anxiety symptoms in the late afternoon

Outcome K6 (≥ 5)	Crude model			Adjusted model		
	OR	95% CI	*p* value	OR	95% CI	*p* value
PA (minutes)	0.99	0.98–1.01	0.294	1.00	0.99–1.01	0.472
K6 level on the previous day	—	—	—	5.10	2.99–8.72	< 0.001
Gender (Women ref: Men)	—	—	—	1.13	0.46–2.78	0.792
Age (≤ 39 years ref: 40–49 years)	—	—	—	3.06	1.09–8.57	0.034
Age (≥ 50 years ref: 40–49 years)	—	—	—	0.36	0.09–1.36	0.133

The generalized linear mixed models analyzed daily-level association across 1400 observation days

*OR* Odds ratio, *CI* confidence interval, *PA* physical activity, *K6* the Kessler Psychological Distress Scale

**Fig. 3:**
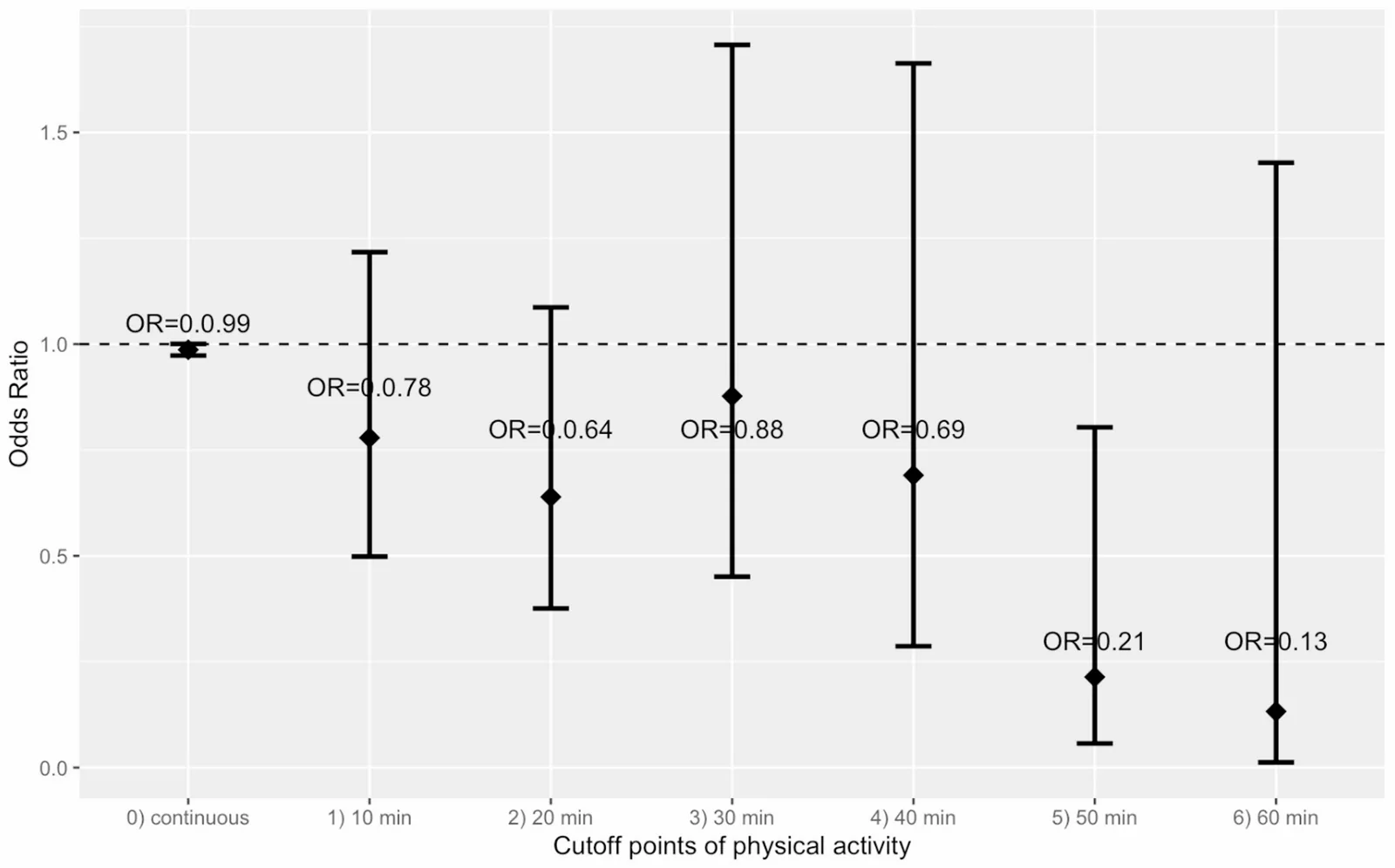
Sensitivity analysis results of physical activity in the early evening (6:00 PM to 9:00 PM)

Table 4 presents the association between early evening physical activity and next-day depression and anxiety symptoms. A negative association was observed in the crude model, though it was not statistically significant (OR = 0.99; 95% CI: 0.99–1.01; *p* = 0.375). In contrast, the adjusted model revealed a marginally significant negative association (OR = 0.99; 95% CI: 0.97–1.00; *p* = 0.061). Regarding the covariates, a higher K6 level on the previous day (OR = 5.46; 95% CI: 3.20–9.31; *p* < 0.001) and younger age (≤ 39 years) (OR = 3.25; 95% CI: 1.23–8.63; *p* = 0.018) were significantly associated with the outcome. Figure 3 illustrates the sensitivity analysis for early evening physical activity. The analysis revealed that only physical activity exceeding 50 min showed statistical significance (OR = 0.21; 95% CI: 0.06–0.80; *p* = 0.022); however, a similar trend was observed in which longer durations of physical activity were associated with lower ORs. In the additional sensitivity analysis that excluded days with extremely high symptom levels (K6 score ≥ 13), engaging in 20 or more minutes of physical activity was significantly associated with a lower risk of next-day depression and anxiety symptoms (OR = 0.54; 95% CI: 0.30–0.99; *p* = 0.046), although the association based on continuous activity duration was not statistically significant (OR = 0.99; 95% CI: 0.97–1.00; *p* = 0.076). The subgroup analysis with the low symptom level (K6 score < 5) on the previous day also demonstrated a significant negative association (OR = 0.98; 95% CI: 0.96–0.997, *p* = 0.023). In this subgroup, physical activity of 20 or more minutes was significantly associated with its lower risk of next-day symptoms (OR = 0.39; 95% CI: 0.18–0.86; *p* = 0.019).

**Table 4:** Multivariate association between physical activity and next-day depression/anxiety symptoms in the early evening

Outcome K6 (≥ 5)	Crude model			Adjusted model		
	OR	95% CI	*p* value	OR	95% CI	*p* value
PA (minutes)	0.99	0.99–1.01	0.375	0.99	0.97–1.00	0.061
K6 level on the previous day	—	—	—	5.46	3.20–9.31	< 0.001
Gender (Women ref: Men)	—	—	—	1.11	0.47–2.60	0.818
Age (≤ 39 years ref: 40–49 years)	—	—	—	3.25	1.23–8.63	0.018
Age (≥ 50 years ref: 40–49 years)	—	—	—	0.39	0.11–1.39	0.148

The generalized linear mixed models analyzed daily-level association across 1400 observation days

*OR* Odds ratio, *CI* confidence interval, *PA* physical activity, *K6* the Kessler Psychological Distress Scale

A post-hoc power analysis was conducted using the adjusted model examining the continuous association between the early evening physical activity and next-day depression and anxiety symptoms. The statistical power of this model was 49% given the current sample size of 1,400 observation days. To achieve 80% statistical power, it was estimated that a total of 3,241 observation days would be required (statistical power = 83.70%).

## Discussion

This study aimed to examine the association between physical activity during specific timeframes and next-day depression and anxiety symptoms among workers. The key finding was that physical activity in the early evening was associated with a lower risk of experiencing a high symptom level for depression and anxiety on the following day, particularly in days with lower prior-day symptom levels (K6 score < 5). Although most analyses did not reach statistical significance to determine an optimal threshold for physical activity duration, a dose-response trend was observed, suggesting that longer durations of physical activity during this timeframe were associated with lower risks of depression and anxiety symptoms. To the best of our knowledge, this is the first study to demonstrate a cross-day association between physical activity and mental health among healthy working adults. These findings may encourage individuals to engage in physical activity as a proactive strategy to prevent mental health conditions and to maintain mental well-being. Moreover, employers and occupational health professionals could incorporate these insights into physical activity recommendations, particularly regarding the timing of activity for optimal mental health benefits.

Our findings are generally consistent with previous studies suggesting that exercise may predict subsequent reductions in depressed mood [15]. Several mechanisms may explain why the early evening was the timeframe in which a significant association emerged among workers. In contrast to the general population, evening time is important for leisure activities for most ordinary workers. One possible explanation relates to participants’ work schedules and daily routines. The original study using the same dataset reported that the absence of an activity peak in the evening was associated with severe-level psychological distress, suggesting that long working hours or high workloads may be contributing factors [24]. Therefore, engaging in physical activity during this timeframe, such as increased walking associated with commuting, may serve as an indicator of a well-regulated daily rhythm in daytime workers. Another study proposed that post-work exercise was associated with a positive effect, and the association was mediated by psychological detachment, sense of belonging, and physical self-perceptions [30]. These findings suggest that a healthy work-life pattern may allow individuals to return home at a reasonable time, recover from work-related stress, and engage in health-promoting behaviors. Another possible mechanism involves improved sleep quality [20]. However, certain direct measurements could not be obtained, such as the actual time to leave the office, types of physical activity, and sleep duration on the index day. In particular, the lack of direct measurement of the actual time to leave the office means that each timeframe may include regular work hours. Therefore, further research is needed to clarify the potential underlying mechanisms.

We were unable to identify a precise duration of physical activity within the observed timeframes that was significantly associated with a lower risk of depression and anxiety symptoms. However, the sensitivity analyses revealed a marked shift in ORs between the 40- and 50-minute cutoff points. In contrast, little variation in ORs was observed for cutoff points below 40 min across both timeframes. These findings suggest that engaging in brief durations of physical activity may be insufficient to produce measurable mental health benefits. Longer durations of physical activity may be necessary to produce an impact on next-day mental health. In the subgroup with the low symptom level (K6 score < 5) on the previous day, a shorter duration, 20 min or more of physical activity, indicated a significant association. Among individuals with low levels of depressive and anxiety symptoms, even modest increases in early evening physical activity may help maintain good mental health.

Several limitations may affect the validity and generalizability of this study. First, the statistical power was insufficient for the analysis. According to the post-hoc power analysis, approximately 2.5 times the current number of observation days would be required to achieve a statistical power of 80% or higher. Second, we could not determine the context of physical activity. Google Fit app does not distinguish between occupational, transport-related, or leisure-time activities. Moreover, physical activity may have been underestimated, as the app only captures activity when the smartphone is carried. Although wearable motion sensors can provide more accurate data [23, 31], we intentionally avoided their use to reduce participant burden and ensure feasibility for large-scale implementation. Third, several potentially important confounding factors were not adjusted for, including basic exercise habit, occupation type, weekly working hours during the previous month, and whether the day was a workday or a non-workday. In addition, individual biological factors such as circadian rhythms may also have influenced the results, particularly if sleep quality served as a mediating factor. Furthermore, we were unable to capture important environmental factors that may influence physical activity, such as ambient temperature and humidity. Finally, the generalizability of the findings is limited. The sample primarily consisted of professional, full-time/regular, day-shift employees [32]. Therefore, the findings may not apply to part-time workers, night-shift workers, or those on rotating shifts.

## Conclusion

In conclusion, physical activity during the early evening is associated with reduced risk of depression and anxiety symptoms on the following day, particularly when prior-day symptom levels were lower. Future studies are warranted to further elucidate the underlying mechanisms by objectively assessing potential mediators.

## Supplementary Information

Supplementary Material 1.

## Data Availability

The data sets generated and analyzed in this study are available with the corresponding author upon reasonable request.

## Abbreviations

K6: the Kessler Psychological Distress Scale
STROBE: the Strengthening the Reporting of Observational Studies in Epidemiology
OR: Odds ratio
CI: Confidence interval
