# Supplementary material for: Association between late afternoon and early evening physical activity and next-day depression and anxiety symptoms among workers in Japan: a two-week longitudinal study
Source: BMC Public Health. 2026 Jun 18;26:2459. doi: 10.1186/s12889-026-28187-2 (PMC13520457; doi:10.1186/s12889-026-28187-2)
Supplement: Supplementary file 1 [file 12889_2026_28187_MOESM1_ESM.docx]

**Appendix 1. The survey form developed in this study**

**Part 1. Enrollment survey**

| **Answer date** | xx/xx/xxxx |
| --- | --- |
| **Name** |  |
| **Company name** |  |
| **E-mail** |  |

**Are you currently on leave from work for any reason?**

- Yes
- No

**Age group**

- <20
- 20-29
- 30-39
- 40-49
- 50-59
- 60-69
- ≥70

**Gender**

- Men
- Women
- Do not answer
- Others

**Occupations**

- Manager
- Professional, engineer, academic
- Clerk
- Sales
- Services
- Transportation
- Construction
- Production/Skilled
- Agriculture/Forestry/Fisheries
- Others

**Weekly working hours (h)**

- 1-34
- 35-40
- 41-50
- 51-60
- 61-65
- 66-70
- ≥71

**Part 2. Daily survey**

| **Answer date** | xx/xx/xxxx |
| --- | --- |
| **Name** |  |
| **E-mail** |  |

**Was today a workday?**

- Workday (a day with work)
- Holiday (a day without work)

The Japanese version of K6

| *Today*, about how often did… | | None of the time | A little of the time | Some of the time | Most of the time | All of the time |
| --- | --- | --- | --- | --- | --- | --- |
| 1 | … you feel nervous? | 0 | 1 | 2 | 3 | 4 |
| 2 | … you feel hopeless? | 0 | 1 | 2 | 3 | 4 |
| 3 | … you feel restless or fidgety? | 0 | 1 | 2 | 3 | 4 |
| 4 | … you feel so depressed that nothing could cheer you up? | 0 | 1 | 2 | 3 | 4 |
| 5 | … you feel that everything was an effort? | 0 | 1 | 2 | 3 | 4 |
| 6 | … you feel worthless? | 0 | 1 | 2 | 3 | 4 |
